# Editorial: Calcium homeostasis and cardiovascular health: mechanisms and therapeutic insights

**DOI:** 10.3389/fendo.2025.1679610

**Published:** 2025-08-20

**Authors:** Ki Ho Park, Bowen Wang

**Affiliations:** ^1^ University of Virginia, Charlottesville, VA, United States; ^2^ Northwestern University, Chicago, IL, United States

**Keywords:** calcium homeostasis, cardiovascular disease, mitochondria-associated membranes (MAMs), vascular calcification, cellular senescence, irisin, atherosclerosis, non-alcoholic fatty liver disease (NAFLD)

Calcium signaling is essential for the regulation of cardiovascular physiology, orchestrating myocardial contraction, vascular tone, gene expression, and more. Disturbances in calcium homeostasis contribute to a wide range of cardiovascular diseases, yet the molecular mechanisms linking calcium dynamics to cardiovascular pathology are not fully understood. In the current Research Topic, we highlight five valuable contributions that uncover new key mechanisms and potential therapeutic targets involved in calcium regulation in cardiovascular health and disease.

In their original research article, Li et al. demonstrated the “*Cardioprotective effect of Yiqi Huoxue decoction on post-myocardial infarction injury mediated by Ca^2+^ flux through MAMs*”. Using both *in vivo* and *in vitro* models, the authors found that Yiqi Huoxue decoction, a traditional Chinese herbal formula, restored mitochondrial-associated membrane (MAM) structure, improved Ca^2+^ homeostasis, and regulated the expression of critical proteins including IP3R2, GRP75, VDAC1, MCU, and Sigma-1R. These findings support a protective role of MAMs in cardiomyocyte injury and open avenues for pharmacological targeting of intracellular Ca^2+^ flux in ischemic heart disease.

Li et al. reviewed the role of “*Endoplasmic reticulum–mitochondria crosstalk: new mechanisms in the development of atherosclerosis*”. This review article comprehensively discussed how MAMs mediate key processes linked to atherogenesis, including lipid metabolism, calcium transport, oxidative stress, and apoptosis. The authors emphasized the dynamic regulation of MAM integrity and suggested that MAM-targeted therapies could be an innovative strategy for modulating plaque progression and cardiovascular risk.

The review by Fang et al. titled “*The functional role of cellular senescence during vascular calcification in chronic kidney disease*” explored how senescent vascular cells promote vascular calcification through the release of microvesicles and senescence-associated secretory phenotype (SASP) factors. These mechanisms enhance osteogenic differentiation in vascular smooth muscle cells (VSMCs) and impair vascular regeneration, suggesting that targeting cellular senescence may represent a promising strategy to mitigate VC in chronic kidney disease (CKD) patients.

Shuangshuang Wang et al. contributed the mini-review “*The emerging roles of irisin in vascular calcification*”, which focused on the exercise-induced myokine irisin. This review highlighted irisin’s protective role in modulating vascular inflammation, endothelial function, and VSMC phenotypic switching, likely via mechanisms involving calcium signaling and oxidative stress. The authors discussed how irisin may serve both as a predictor and a potential therapeutic modulator for vascular calcification and hypertension.

Finally, the original research article by Hao et al. titled “*Observational and genetic association of non-alcoholic fatty liver disease and calcific aortic valve disease*” identified an association between non-alcoholic fatty liver disease (NAFLD) and increased risk of calcific aortic valve disease. =The link to ectopic calcification and systemic metabolic dysfunction provides critical context for calcium-driven cardiovascular pathologies.

Together, these articles reflect the complex, multi-layered role of calcium homeostasis in cardiovascular disease.

We thank all the contributing authors for their valuable insights and efforts. We hope this Research Topic will inspire further exploration into calcium-centered mechanisms and therapeutic innovation in cardiovascular research.

